# Comparing vaccination coverage and dog population demographics among four pilot dog rabies vaccination strategies in Uganda

**DOI:** 10.3389/fvets.2025.1656563

**Published:** 2025-10-13

**Authors:** Dickson Akankwatsa, Terence Odoch, Anna Mary Kahunde, Sonja Hartnack, Arthur Bagonza, Juliet Kiguli, Samuel George Okech, Adrian Herrera, Clovice Kankya, Lamorde Mohammed, Doreen Agado, Monique Léchenne, Frederic Lohr, Andrew Kambugu, Salome Dürr

**Affiliations:** ^1^Department of Epidemiology and Biostatistics, School of Public Health, Makerere University College of Health Sciences, Kampala, Uganda; ^2^Department of Global Health Security, Infectious Diseases Institute, Makerere University, Kampala, Uganda; ^3^Department of Biosecurity, Ecosystems and Veterinary Public Health, College of Veterinary Medicine, Animal Resources and Biosecurity, Makerere University, Kampala, Uganda; ^4^Department of Production Kyegegwa District Local Government, Kyegegwa, Uganda; ^5^Section of Epidemiology, Vetsuisse Faculty, University of Zurich, Zurich, Switzerland; ^6^Department of Community Health and Behavioral Sciences, School of Public Health, Makerere University College of Health Sciences, Kampala, Uganda; ^7^Department of Veterinary Pharmacy and Clinical Studies, School of Veterinary Medicine and Animal Resources, Makerere University, Kampala, Uganda; ^8^Veterinary Public Health Institute, Vetsuisse Faculty, University of Bern, Bern, Switzerland; ^9^Swiss Tropical and Public Health Institute, Allschwil, Switzerland; ^10^University of Basel, Basel, Switzerland; ^11^Worldwide Veterinary Service, Cranborne, United Kingdom

**Keywords:** zero by 30, integrated vaccination campaign, Bayesian model, dog population estimate, rabies control, dog vaccination campaigns

## Abstract

**Introduction:**

The zero by 30 initiative aims to eliminate dog-mediated human rabies by 2030, for which dog vaccination is a crucial pillar. This study piloted four different dog vaccination campaign strategies in Kyegegwa, a rural district in Uganda, where rabies is endemic, and compared the vaccination coverages achieved by the strategies.

**Methods:**

Four vaccination strategies were rolled out, each in three parishes from different sub-counties: (i) static point vaccination (SP), (ii) school-based (SB, i.e., information campaigns were mainly conducted at schools and vaccination was done at the school during weekends), (iii) integrated dog with livestock vaccination (D-L), and (iv) integrated dog vaccination with human health services (D-H, One Health approach). Vaccination coverage was estimated using transect and household survey data, analyzed with a Bayesian model that estimated, besides the vaccination coverage, the dog population size and the proportion of ownerless dogs for each dog population.

**Results:**

The mean vaccination coverage achieved among the owned dog population across the three parishes for each respective strategy was 29.5% for SP strategy (the model converged in one parish only), 53.9% (range 27.4–79.5%) for SB, 66.2% (range 53.5 and 86.0%) for the D-L, and 74.5% (range 63.7 and 88.4%) for D-H. The mean proportion of ownerless dogs in the villages investigated was estimated at 0.1% for the parishes with SP strategy, 7.0% (range 0.1–20.8%) for SB strategy, 29.7% (range 0.5–88.1%) for D-L, and 7.9% (range 0.3–17.7%) for D-H strategy villages.

**Discussion:**

The strategy integrating dog vaccination with human health services outperformed the other strategies by achieving the highest mean vaccination coverage and reaching a constantly high coverage of above 60% for all the three parishes of that strategy. This demonstrates the potential of the human-animal integrated D-H vaccination strategy as an effective approach for rabies control. Sensitization strategies for dog owners also depended in the vaccination strategy performed, i.e., spread of information through health centers for the D-H strategy, which is part of the success of this strategy. The study needs to be taken as a pilot, because of limitations such as different settings between the sub-counties. Further testing across diverse settings can help assess integrated dog vaccination strategies’ consistency and scalability, providing valuable insights for developing a One Health model to strengthen future rabies elimination efforts.

## Introduction

Rabies is a viral zoonotic disease that causes progressive encephalitis and leads to death once symptoms occur (1). Globally, rabies is estimated to cause approximately 59,000 human deaths annually, of which 99% are dog-mediated (2). The World Health Organization (WHO) estimates that about 40% of rabies deaths occur in children below 15 years (3). In Uganda, 14,865 animal bites were officially recorded between 2015–2020, 90% of which were bites from dogs (4). In addition, 36 human deaths from suspected rabies were recorded during the same period (4). Most likely, as in other regions, these records are largely underreported. Historical surveillance data between 2001 and 2015, shows that most exposures in Uganda occur in the Central and Northern regions, where each accounts for 27% of reported animal bite injuries, with the Northern region also recording the highest bite incidence of 76 per 100,000 population (5).

In 2015, the WHO, the World Organization for Animal Health (WOAH), the Food and Agriculture Organization of the United Nations (FAO) and the Global Alliance for Rabies control (GARC) launched the “zero by 30” goal to eliminate dog-mediated human deaths by 2030 (6). Public awareness on rabies, post-exposure prophylaxis (PEP) in humans after exposure to a suspected rabid animal, and dog mass vaccination are crucial pillars of the strategy to reach this goal (7–9). Empirical and simulation studies in several regions, including Africa, have repeatedly shown that a 70% vaccination coverage of dogs is effective for the elimination of rabies from canine populations (10–12). However several studies have reported failure in achieving such vaccination coverage, especially in Sub-Saharan Africa, caused by lack of availability and affordability of vaccines, low political priority, and technological challenges (12–15).

Most dog vaccination campaigns use static points or door-to-door strategies. Some studies have demonstrated that door-to-door strategy can reach a higher coverage, however it demands substantial logistical effort and is expensive to roll out (16, 17). Development and testing other approaches to supplement these vaccination campaigns are expected to increase vaccination attendance (16). Earlier studies in Chad tested combined animal and human vaccination approach among nomadic pastoralists for diseases other than rabies (18, 19). These campaigns revealed that the approach was successful regarding vaccination coverage in both humans and animals, and less expensive as compared to separate interventions. In addition, it was shown that the integrated approach better involved community members, which increased the level of trust for both, vaccination of children and livestock (18, 20). Similarly, a study in Tanzania revealed lower costs for integrated health delivery services, such as anthelmintic treatments for school children combined with rabies vaccination in dogs, compared to offering the two services separately (19). Integrating vaccination campaigns between dogs and livestock has also been discussed. For example, the WOAH strongly encourage countries to implement vaccination programs against Peste des Petits Ruminants (PPR) and rabies (20). It is important to explore the potential for integration of these vaccinations.

In Uganda, where nearly 90% of the population reside in rabies endemic communities (21), dog vaccination campaigns against rabies use static or DTD strategies (4, 17), and have been conducted inconsistently across districts depending on districts’ capacities and logistical hindrances and with varying vaccination coverages reached (21–23). In 2022, the country validated its National Rabies Elimination Strategy (2022–2030), adopting a One Health approach in line with the global “Zero by 30″ goal. Key components include annual mass dog vaccination campaigns, improved access to PEP, strengthened rabies surveillance and diagnostics, and community awareness initiatives (24). The Ministry of Animal, Agriculture, Industry and Fisheries (MAAIF) procures approximately 500,000 rabies vaccine doses annually for dogs and distributes them to districts on request free of charge. However, the country wide dog vaccination coverage was estimated as low as 10% (4), attributed to operational challenges, limited resources, low political prioritization, distances involved and low public awareness and demand, depending in the region (4, 17). Integrated strategies as used in other countries may also be successful to increase vaccination coverage for rabies in Uganda.

In the present study, we aimed at piloting four rabies vaccination campaign strategies for dogs in Kyegegwa district in rural Uganda: the static point (SP) strategy, plus three more innovative strategies, i.e., school-based (SB), integrated dog with livestock vaccination (D-L) and integrated dog vaccination with human health services (D-H). We compared the success of the pilot vaccination strategies by estimating the coverage reached. In addition, the methodology applied allowed us to estimate the dog population size and the proportion of ownerless dogs in the villages under investigation.

## Materials and methods

### Study area and setting

The study was conducted in Kyegegwa district, a rural area located in the Mid-Western part of Uganda. According to the last national population and housing census conducted in 2024, the district has approximately 501,000 inhabitants (25). Most inhabitants across the district carry out economic activities of mixed farming and small-scale trading and have a range of cultural and religious backgrounds. There is no published data on dog population and rabies situation in the district, however unpublished estimates from the district veterinary office indicated that the district has approximately 25,000 dogs with about 300 dogs per parish and 25 dogs per village. Additionally, approximately three suspected rabies dog bites are reported every month to the veterinary office, some of which human victims succumb to rabies.

**Figure 1:**
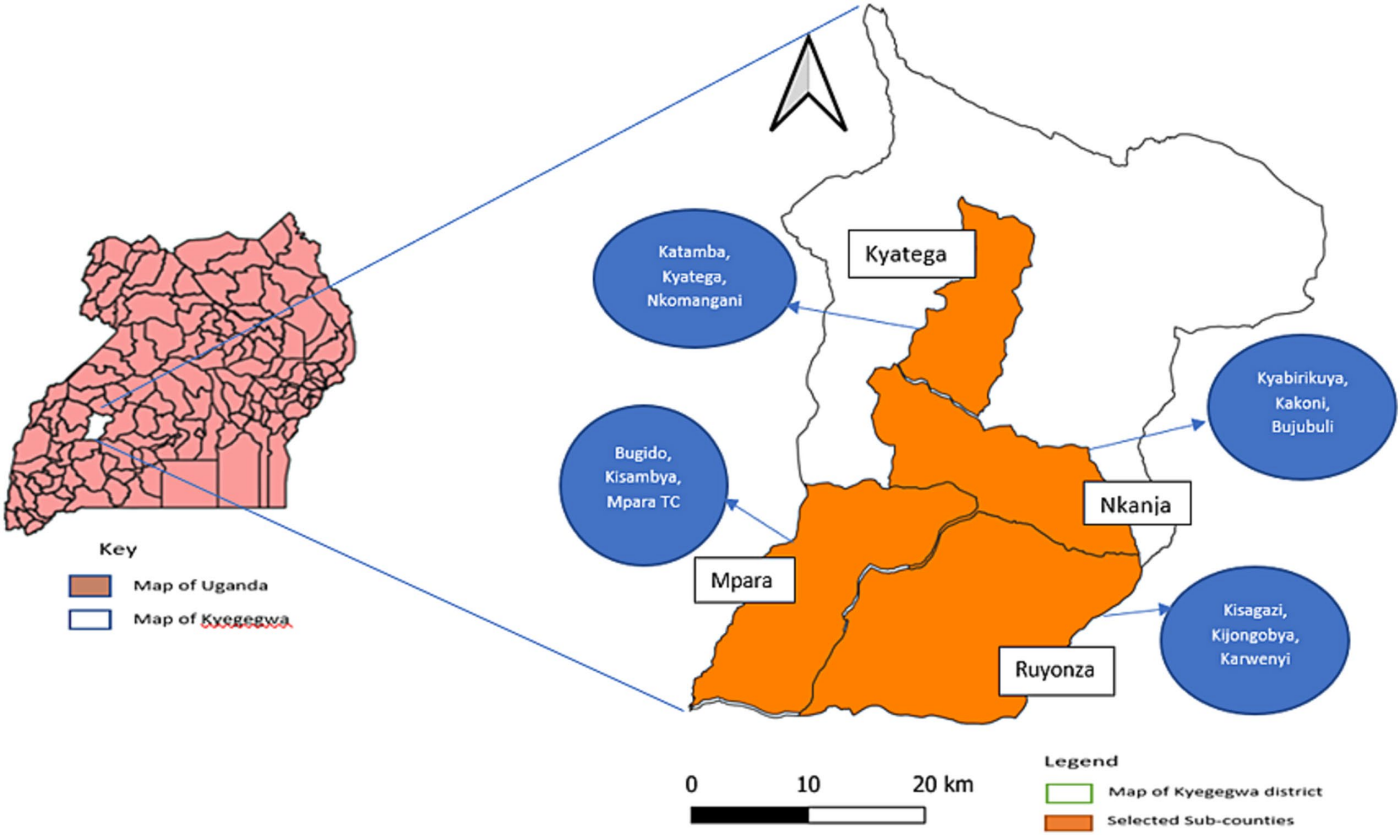
A map of Uganda showing the four selected sub-counties with the names of the 12 selected parishes in Kyegegwa district for a dog vaccination campaign study against rabies in 2023.

The study was conducted between March and September 2023 in 12 parishes from four sub-counties, i.e., Nkanja, Ruyonza, Kyatega and Mpara Town Council. Nkanja has two public health facilities (26), with Bujubuli HCIII as a major health facility that has an active community health outreach program for human health services such as immunization, serving close to 24% of the refugees in Kyaka II (27). Bujubuli HCIII is located in Bujubuli parish, which was included in our study with a large proportion of refugees. Ruyonza is located in the cattle corridor of the district, and predominantly occupied by the Bahima tribe, who mainly are cattle keepers for livelihood. Kyatega has many schools including 17 primary and one secondary schools (28, 29). Mpara Town Council has mixed background, urban and peri-urban. Therefore, the sub-counties were purposively selected based on either having a high number of schools (Kyatega), livestock (Ruyonza), reported rabies cases (Mpara Town Council), or public health facilities with active community outreaches (Nkanja), respectively, linked to the four campaign strategies to be investigated. The three parishes within each sub-county were randomly selected (Figure 1).

### Four dog vaccination campaign strategies

Four different dog vaccination strategies were piloted in the 12 selected parishes, namely (i) static point vaccination (SP), (ii) school-based vaccination (SB), (iii) integrated dog with livestock vaccination (D-L) and (iv) integrated dog vaccination with human health services (D-H). Each of the strategies was conducted in the selected three parishes of the same sub-county. The decision to implement a specific strategy in a sub-county, as well as the selection of the specific study sites and vaccination points in each parish, was preceded by consultative discussions with key stakeholders, including representatives from the District Health Office, District Veterinary Office, District Education Office and the community. The message of dog vaccination to the public was first announced at the launch event for the dog vaccination in Kyegegwa town in March 2023, followed by a radio announcement diffused within the entire district.

The SP strategy was conducted in Mpara Town Council at five vaccination points in March–April 2023 during weekdays (Supplementary Table S1). Dogs were brought for vaccination by dog owners (adults or children) to a static vaccination point that was defined by the local authorities. Mobilization and information sharing about vaccination were done through the local council chairpersons, churches and house-to-house three to 5 days in advance of the campaign.

The SB strategy was conducted in Kyatega subcounty at 17 vaccination points, in June 2023 (Supplementary Table S1). Sensitization on rabies and its prevention was conducted in schools by the research team. The researchers shared the vaccination schedule with the teachers and the teachers shared the schedule with pupils and asked them to communicate to their parents and guardians. In addition, the researchers shared the schedule with the local council chairperson, and community health workers who supported the mobilization and sensitization. On the vaccination day, either parents or pupils brought the dogs to the vaccination point. This strategy was organized only on weekends when the school children had no classes.

The D-L strategy was conducted in Ruyonza sub-county at eight vaccination points in March–April 2023 during weekdays (Supplementary Table S1). Dog vaccination was carried out at the time when routine livestock vaccinations were conducted, for example, against foot and mouth disease (FMD) or Peste des Petits Ruminants (PPR). Sub-county veterinary officers were contacted and the livestock vaccination schedule consulted. Upon reaching consensus to integrate dog and livestock vaccination, the area veterinary officer communicated to the dog and livestock owners through the local council chairpersons the days and farms of integrated livestock (PPR or FMD) and dog (rabies) vaccination. Dog owners in the surroundings brought the dogs close to the respective farms.

The D-H strategy was conducted in Nkanja sub-county at 13 vaccination points in June–July 2023 during weekdays (Supplementary Table S1). Dog vaccination campaigns were carried out at the time and place where the healthcare workers conducted routine health services like children’s immunizations, nutrition assessments, and health sensitizations campaigns. Bujubuli Health Centre III, which offers health services in the parishes involved was consulted and dates of outreach health services were obtained. The schedule of the children vaccination planned in the selected parishes was shared with the local council chairpersons and village health teams. They then mobilized the dog owners by door-to-door information to participate in the vaccination campaigns, and using posters at places of convergences like in trading centers and at churches. In addition, the healthcare workers of the health center supported in the mobilization by communicating the upcoming dog vaccination to the communities during their outreach activities. On the vaccination day, the dogs were brought to sites nearest to the human health services outreach site.

### Organization of the vaccination campaigns

The vaccination team comprised of one veterinarian from the respective sub-county, employed by the Kyegegwa district local government, vaccinating the dogs and issuing the certificates. In addition, a member of the research team collected data and marked dogs, and a site-specific community mobilizer supported communication.

The vaccination campaigns targeted dogs, however in case a cat was presented, it was also vaccinated. The vaccination was free of charge for the owners. All animals 3 months and older were considered for vaccination. Each animal was registered to capture information on species, name, sex, age, village of residence, owner’s name, rabies vaccination history, number of dogs the person keeps, and age category of the person brought the animal for vaccination. The information was recorded digitally using the Worldwide Veterinary Service (WVS) application,^1^ and on hard paper by the district personnel for purposes of record keeping and accountability. Each dog received 1 mL of Nobivac® vaccine subcutaneously around the neck or the thigh. A nose-mouth muzzle was used if needed on the dog during vaccination to protect the vaccinator and dog handler from bites. Nonetheless, an emergency safety kit was present. The vaccines were obtained through the district vaccine requisition system, kept at the district in the vaccine fridges, and were carried to the vaccination site using vaccine carriers which contained ice packs to maintain temperatures between +2 and +8 °C.

Upon vaccination, each dog was marked on the head, neck and on flanks using an animal marking crayon for easy identification during post-vaccination surveys. A certificate indicating the date for the next vaccination, with dog and owner details, including a barcode for the vaccine was offered to the owner for each vaccinated animal. The campaign was supervised by district officials and a team of researchers who ensured that supply of consumables such as, syringes, vaccines, nose muzzles and crayon markers, was done timely.

### Post vaccination data collection

To determine the vaccination coverage, estimation of the total dog population, and proportions of ownerless dogs in all the surveyed sites, a capture-recapture method as previously piloted by Kayali et al. in N’Djamena (30), and later used to evaluate coverage of large-scale mass-vaccination in the same town (14) was used. This approach combines two datasets, one collected during transect walks, and one during a household survey. Villages with at least five dogs vaccinated during the campaigns were selected for the surveys.

Two teams each comprising of one to two researchers and one local official walked along the predefined transect lines in the morning from 08:00–10:00 h and evening from 16:00 to 18:00 h the day after the vaccination campaign. Using the WVS application, the area to be covered by the transect was recorded as a polygon, and transect lines were drawn with the polygon following main routes as well as in routes passing through areas with concentrated households (Supplementary Figure S1). The proportion of the polygon area covered by observation was calculated by dividing the total length of the transection lines times 100 m (assuming that observation was done 50 m to each side of the transect line) by the area of the polygon. The two teams started from the start and end point in the transect line, respectively, moving in opposite directions. During the walks, they counted every dog seen and noted whether the dog had a mark (implying vaccinated) or not (implying unvaccinated), and whether the dog was sighted on the street or in the compound, providing insights regarding dog confinement. This data, together with the actually walked paths, was captured digitally using the transect survey module of the WVS application.

On the same day of the transect walks, a household survey was conducted in all households of the respective village. The following information was collected during interviews that lasted about 10 min each: whether people keep dogs, how many dogs are kept, how many dogs they had vaccinated during the current vaccination campaign, reasons for non-vaccination, whether their dogs roamed or were always confined, and if they see unowned dogs around their homes. The questionnaire was written in English, and where needed, translated orally during the interview into the local language Rutooro. This data was captured digitally in the household survey module of the WVS application.

### Data management and analysis

To ensure data quality, data was checked for accuracy, completeness, and consistency on the evening of each fieldwork day, and corrected if needed. The data from the WVS application was downloaded in comma-separated value (CSV) format. Descriptive statistics were performed on the vaccinated dogs and data collected during the transect and household surveys to describe the study population reached by vaccination. R version 4.4.1 was used for the descriptive analysis (31).

A Bayesian model as previously used in Chad was applied to estimate the vaccination coverage amongst the dog population, the total dog population size, and the proportion of owned to unowned dogs (30, 32) (Supplementary material S1). Prior information on the confinement probabilities for owned and unowned dogs, as well as recapture probabilities of dogs during the transects was assumed to be the same for all villages. Values of the prior information for dog confinement as well as for the proportion of ownerless dogs were taken from an earlier conducted study in Chad (32) (Supplementary Table S2). The model analysis was conducted at parish level, by summing up the data collected during the transect walks and household surveys from the villages in the respective parish. The data included into the model for a parish consisted of the total number of dogs vaccinated within the villages investigated in the respective parish, the numbers of marked and unmarked dogs collected, separated for the morning and evening transect walks, as well as the number of vaccinated and unvaccinated owned dogs recorded during the household survey (Table 1), and their confinement rate.

**Table 1:** Number of vaccinated dogs during dog vaccination campaigns in 12 parishes in Kyegegwa district, Uganda, the number of marked (i.e., vaccinated) and unmarked (i.e., unvaccinated) dogs observed during transect walks, and dogs found in households the day after the vaccination campaigns.

Subcounty and Parish	No. dogs vaccinated*	Morning transect route		Evening transect route		Household survey	
		No. of marked dogs	No. of unmarked dogs	No. of marked dogs	No. of unmarked dogs	No. marked dogs in the households	No. unmarked dogs in the households
Mpara town council: static point (SP)							
Mpara central	8	0	27	3	17	1	4
Kisambya	7	6	3	0	1	11	1
Bugido	39	13	7	3	9	13	10
Kyatega: School-based (SB)							
Kyatega	124	3	7	2	16	14	40
Nkomangani	95	14	2	11	0	7	7
Katamba	44	6	7	4	5	12	3
Ruyonza: integrated dog with livestock vaccination strategies: D-L							
Kisagazi	18	9	7	2	2	4	3
Karwenyi	29	0	2	9	2	7	1
Kijongobya	42	9	35	0	0	13	12
Nkanja: integrated dog vaccination with human health services: D-H							
Bujubuli	159	14	16	17	16	37	15
Kakoni	33	1	4	0	0	9	1
Kyabirikuya	28	4	6	8	6	12	7
Total	626	79	123	59	74	140	104

*Indicates the number of dogs vaccinated in the villages where the transect walks and household surveys were conducted.

The model was implemented in OpenBUGS software.^2^ For each analysis at parish level, three chains of the simulation were run independently for 100,000 iterations after an initial burn-in of 50,000 iterations and a thinning of 10 iterations, leading to a sample of 300,000 iterations. The posterior values of the outcome variables together with 95% credibility intervals were obtained using Markov chain Monte Carlo simulation (33). Outcome variables contained, on parish level, the vaccination coverage amongst the entire dog population and amongst the owned dog population, the dog population size, as well as the proportion of ownerless dogs in the observed dog population in the streets. A sensitivity analysis was performed to investigate the influence of the prior values on the posterior estimates. One parish was chosen randomly from each of the four strategies and the priors for the uniform distribution (pmin-pmax) for the recapture probabilities during transect (originally pmin = 0.056; pmax = 0.54) were varied with values of 0.01 and 0.1 for pmin and 0.6, 0.8 and 0.99 for pmax, resulting in the six combinations of pmin-pmax of (0.01/0.6 0.01/0.8, 0.01/0.99,0.1/0.6, 0.1/0.8, 0.1/0.99). Similarly, the prior values for the means (m1, m2) and standard deviations (s1, s2) of the confinement probability of the marked dogs (originally m1 = 0.286, s1 = 0.1) and the unmarked dogs (originally m2 = 0.196, s2 = 0.1) were varied with values of (m1/s1 with 0.1/0.1, 0.3/0.2 and 0.5/0.2).

### Ethical approval and consent to participate

The study was approved by the Uganda National Council for Science and Technology (UNCST) (HS3463ES) and the Makerere University School of Public Health Research Ethics Committee (MakSPH-REC, approval number 187). Additionally, official permission for data collection at the district and parish levels was obtained from the responsible authorities.

## Results

### Descriptive statistics of study population

#### Animals vaccinated during the vaccination campaigns

In total, 890 animals were vaccinated, 850 (95.5%) dogs and 40 (4.5%) cats, out of which 552 (62.0%) were males, 298 (33.5%) were females, and for 40 (4.5%) sex was not recorded. All 890 animals were 3 months old or above (according to the owners’ reports) at the time of vaccination. Of the females, 230 (77.2%) were non-pregnant, 34 (11.4%) were pregnant, 30 (10.1%) lactating, and 4 (1.3%) had unknown pregnancy status (owners were not sure and no visible signs of pregnancy noted during vaccination). In total, 116 (13.0%) of the animals were brought to the vaccination sites by children aged 15 years and below, while 768 (86.3%) were brought by adults above 15 years, and for 6 owners (0.7%) their age bracket was not recorded. Ninety-eight (11.5%) of the dogs presented to the vaccination site had been previously vaccinated against rabies, 702 (82.6%) had never been vaccinated, while the previous vaccination status of 50 (5.9%) dogs was unknown according to the owners.

The number of dogs vaccinated during the SP strategy was 152 in five sites (Supplementary Table S1). In the SB strategy, 17 sites were used reaching a total of 292 vaccinated dogs. During the D-L strategy, 124 dogs were vaccinated in 8 sites, and in the D-H strategy 282 dogs were vaccinated in 13 sites.

#### Dogs observed during transect walks and household survey

The villages that contributed more than five dogs at vaccination sites for vaccination were counted and included in the survey as follows; 7/24 (29.2%) for the SP strategy, 9/16 (56.3%) for the SB, 7/12 (58.3%) for the D-L, and 15/22 (68.2%) for the D-H strategy. In total, 626 (73.6% of all dogs) were vaccinated within the villages that were used to estimate vaccination coverage, i.e., where the transects and household surveys were conducted (Table 1).

The proportion of the area covered by the transect walks ranged between 1 and 100% (median 33%, interquartile range 26–50%). The number of total dogs sighted during the morning transect walks (*n* = 202) was higher than during the evening transect walks (*n* = 133). Among the 335 dog sightings, 138 (41.2%) were marked, indicating they had been vaccinated during the performed study, while 197 (58.8%) were unmarked. Notably, a larger number of dog sightings (179, 53.4%) were observed within household compounds, compared to the 156 sightings (46.6%) recorded on the streets (Supplementary Table S3).

A total of 608 households were visited in the household survey, with 153 (25.2%) of them keeping dogs. The proportion of dog-owning households is higher in Mpara Town Council compared to the other sub-counties, which have a comparable proportion of households with dogs (Table 2). In total, 274 dogs were found in these households, leading to a mean of 1.82 dogs per dog keeping households (median = 1.0, min = 1, max = 9), although data on the number of dogs kept from 25 owners was missing. The number of dogs per dog-owning household is similar for all sub-counties (Table 2). The overall dog:human ratio within the interviewed households was estimated at 1:3.4 (95% C. I. 1:3.1–1:3.8), and differed between sub-counties, with a decreasing ratio from Kyatega, Nkanja, Ruyonza to Mpara Town council (Table 2). Amongst the 274 dogs found in the households, 140 (57.4%) were marked and 104 (42.6%) were not marked during the household survey (Table 1).

**Table 2:** Comparison of dog keeping characteristics between the four sub-counties involved in a dogs vaccination study in the district of Kyegegwa, Uganda.

Sub-county	No. households	No. households with dogs	Proportion of households with dogs (estimate, 95% C. I,)	Median (min-max) no. dogs per Household	Human population in households	Dog population in households	Dog-human ratio estimate (95% C. I.)
Nkanja	164	37	22.6 (16.5–30.0)	2 (1–6)	216	81	1:2.7 (1:2.3–1:3.2)
Mpara town council	160	59	36.9 (29.5–44.9)	1 (1–6)	380	60	1:6.3 (1:5.0–1:8.1)
Ruyonza	159	29	18.2 (12.7–25.3)	2 (1–8)	198	54	1:3.7 (1:2.9–1:4.7)
Kyatega	125	28	22.4 (15.6–30.9)	2 (1–9)	153	83	1:1.8 (1:1.6–1:2.2)

In 85 (55.6%) of the households which had dogs, some or all dogs were not taken for vaccination. Several reasons for non-participation were indicated: 21 (24.7%) reported that they had not been informed about their campaign, five (5.9%) had no means of transport, five (5.9%) had very young puppies, and three (3.5%) had too aggressive dogs. However, the majority (48.2%, *n* = 41) did not mention any reasons for not taking their dogs for vaccination.

### Estimation of vaccination coverage and dog population size

The dog vaccination coverage amongst the owned dog population across the parishes ranged between 27.2% in Kyatega, Kyatega sub-county applying the SB strategy, and 83.8% in Karwenyi, Ruyonza sub-county, applying the D-L strategy (Table 3). The proportion of ownerless dogs across parishes ranged between 0.1% in Kyatega, Kyatega sub-county, and 81.1% in Kijongobya, Ruyonza sub-county. This reduced the vaccination coverage amongst the entire dog population to 27.2% in Kyatega subcounty (SB) to 83.8% in Karwenyi subcounty (D-L). The dog population size per parish was estimated between 30 dogs in Kisagasi, Ruyonza sub-county, and 453 dogs in Kyatega, Kyatega sub-county (Table 3). In Mpara Town Council and Kisambya parishes, the Bayesian model did not converge, because the number of vaccinated dogs found during the household surveys was higher than the number of vaccinated dogs recorded during the campaign. Thus, only data from Bugido parish is presented.

**Table 3:** Estimates of the overall dog vaccination coverage, vaccination coverage amongst the owned dogs, dog population size, and proportion of ownerless dogs amongst 10 parishes in four sub-counties in Kyegegwa district, using four different strategies.

Sub-country (strategy) Parish	Overall vaccination coverage (%)	Vaccination coverage amongst owned dogs (%)	Total dog population size estimate	Percentage of ownerless dogs in dog population (%)
	Estimate (95% credibility interval)	Estimate (95% credibility interval)	Estimate (95% credibility interval)	Estimate (95% credibility interval)
Static point (SP)*				
Bugido	29.3 (26.2–32.9)	29.5 (26.6–33.0)	132.4 (118.1–146.8)	0.1 (0–5.7)
Mean	29.3	29.5	132.4	0.1
Kyatega (School-based: SB)				
Nkomangani	54.6 (50.7–59)	54.8 (51–59.1)	173.4 (160.7–186.1)	0.1 (0–3.3)
Katamba	66.3 (42.4–79.3)	79.5 (72.9–87.9)	55.4 (50.1–60.4)	20.8 (0–96.1)
Kyatega	27.2 (25.6–28.5)	27.4 (26.3–28.6)	452.6 (433.2–471.8)	0.1 (0–5.9)
Mean	49.4	53.9	227.1	7.0
Ruyonza (integrated dog with livestock vaccination strategies: D-L)				
Kijongobya	29 (25.2–38.9)	53.5 (49.0–58.8)	78.5 (71.4–85.8)	88.1 (35.4–100)
Kisagazi	57.5 (48.2–65.6)	59.2(51.7–68.9)	30.4 (26.1–34.8)	0.5 (0–27.0)
Karwenyi	83.8 (71.8–91.2)	86 (78.6–94.9)	33.7 (30.6–36.9)	0.6 (0–22.8)
Mean	56.8	66.2	47.5	29.7
Nkanja (integrated dog vaccination with human health services: D-H)				
Bujubuli	67.3 (50.9–72.9)	71.3 (68.7–74.2)	222.9 (214.4–231.3)	5.7 (0–40.9)
Kakoni	75.7 (46.7–87.5)	88.4 (80.8–98.7)	37.3 (33.4–40.9)	17.7 (0–98.4)
Kyabirikuya	62.4 (52.8–69.5)	63.7 (57.5–70.8)	44 (39.6–48.7)	0.3 (0–21.7)
Mean	68.5	74.5	101.4	7.9

*The Bayesian model generated errors for the two parishes Mpara Central and Kisambya in Mpara Town council, the reason why estimates from only Bugido parish is presented.

**Figure 2:**
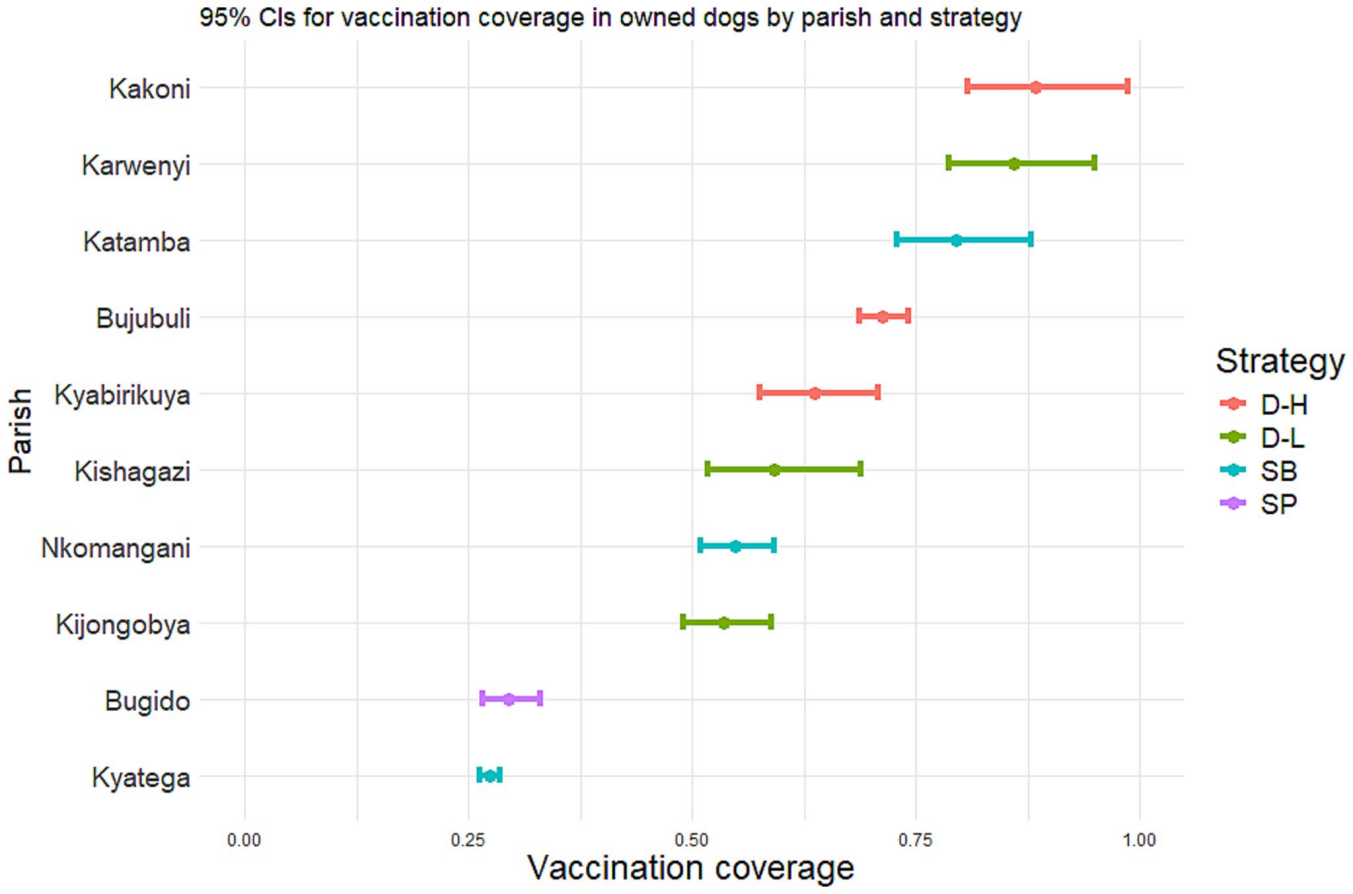
Estimates and 95% credibility intervals for the vaccination coverage reached among owned dogs by four different campaign strategies in four sub-counties in Kyegegwa district, Uganda.

On average, the D-H strategy reached the highest vaccination coverage among owned dog population (74.5%), followed by D-L (66.2%) (Table 3). When comparing the different vaccination campaign strategies across the parishes, the D-H strategy was the only strategy with vaccination coverages of above 60% in all the three parishes (Figure 2). The SB reached coverage above 60% in only one parish, while the coverage was moderate to low in the two other parishes. The D-L strategy reached a very high vaccination coverage in one parish, while for the other two less than 60% was achieved. The SP generally had the lowest coverage for the one parish. A vaccination coverage of above 50% was reached in all but two parishes.

### Sensitivity analysis

Sensitivity analysis for the priors for recapture probabilities (pmin, pmax) in the transect survey was performed by decreasing pmin and/or increasing pmax relative to the original priors. The largest differences – in terms of percentage difference to the original posterior estimates—were found for one parish (Karwenyi) in the total vaccination coverage with 1.17 and 1.22% for pmax values of 0.8 and 0.9, respectively. All other posterior percentage differences were less than 1%.

When the priors (mean and standard deviation) for the confinement probabilities of marked (owned) dogs were varied, the largest percentage difference in the posterior estimates of total vaccination coverage was 1.67%, observed when the prior mean was decreased to 0.1, and the standard deviation was 0.1. All other percentage differences from the initial posterior estimates were below 1%.

Varying the priors for the confinement probability of unmarked (unowned) dogs—specifically by increasing the mean values to 0.3 and 0.5 and the standard deviation to 0.2—resulted in the largest percentage differences of a maximum of 2.82% in the posterior estimates of total vaccination coverage.

Taken together, the sensitivity analysis gives evidence that – if the model converges – the priors have little impact on the posterior estimates.

## Discussion

In this study, four strategies of dog vaccination campaigns against rabies were tested in rural Uganda.

Among the four strategies, the integrated approach involving dog vaccination and human health services achieved, as the only strategy, a consistently high coverage. One element of this success may be the mobilization tactics employed during its implementation. Human healthcare workers played a crucial role by integrating the message on dog vaccination while they reminded the mothers and children for the routine immunization. They communicated that while the mothers brought their children for immunization, they could take advantage and bring their dogs too for vaccination by the veterinary teams who would be around at the same location. Interestingly, many mothers considered bringing their children first, and then went back to get their dogs, possibly reflecting fears around the aggressiveness of dogs. This had been mentioned by participants during the vaccination campaigns. Another positive effect on the vaccination coverage achieved may be the perceived value of the dogs in this area. A part of the D-H strategy (Bujubuli Parish) was rolled out in a refugee setting where dogs were highly valued for hunting, security, and even as a source of food. This is a contrasting finding as established by other studies such as by Mbaipago et al. in Chad, which found participating in dog vaccination low in communities where dogs were a source of food, for reasons linked to the therapeutic effects of vaccine on dog meat (34). Chevalier et al. also reported higher vaccination coverage in dogs in communities where people value their dogs (35). Furthermore, the high performance of the D-H strategy could be linked to the organized and command-oriented leadership within the settlement. The chief commandant of the community helped to disseminate information about the campaigns, enhancing community responsiveness. Although the use letters and notices, and information of local councils and church leaders were considered as communication strategies, this community’s prior experience in mobilization prompted a more grassroot approach such as house to house announcements, and use of megaphones that were readily used during our campaign, a method also used successfully in a suburban community in Uganda (36). Future campaigns should consider varied campaign communication strategies to ensure sufficient reachability of vaccination messages to dog owners.

The high vaccination coverage achieved for the integrated D-H strategy in this study resonate with findings in other studies. For instance, Lankester et al. found strong community support and high participation during integrated campaigns combining community wide mass drug administration targeting soil transmitted helminths and vaccination of dogs against rabies (19). High vaccination coverage can also be attributed to prior stakeholder engagements regarding their preferences on the strategies for roll out. During this study, an exchange with stakeholders was performed to commonly define which of the four strategies to apply. This observation is supported by a related study in Chad that recommended prior planning for joint campaigns to identify in advance potential services access barriers, that can be considered ahead of the roll out of interventions (37).

The D-L achieved high vaccination coverage only in one parish, and a moderate coverage in the other two parishes. A reason for the only moderate coverage could be linked to the fact that it was rolled out mainly in the cattle corridor of Kyegegwa district, which is predominantly occupied by the Bahima tribe. We learnt from the stakeholders in discussions after the campaign that this tribe are established cattle keepers, who attach a high economic value on their livestock, and not on dogs. This perspective is not unique to Uganda. A study by Belshaw et al. in United Kingdom also noted that participants were less interested in vaccinating dogs because of their lack of monetary value (38). Similarly, in Zambia, low participation in vaccination of dogs was equally attributed to the fact that dogs did not attract any monetary value (39). On the other hand, as argued by Hauskeller, the value of dogs should not only be taken when they serve a purpose to humans but rather when they have a purpose for other animals, in addition to their intrinsic value (40). Therefore, it is important to consider broader aspects of the importance of dogs to shape future vaccination campaigns planning.

Another aspect that was raised during conversation with stakeholders after the vaccination campaigns was the risk of spreading parasitic diseases from dogs to livestock. Indeed, some parasites, such as cestodes, perform a herbivore-carnivore cycle, so that biosecurity issues arise when dog feces are brought in contact with livestock pasture.

The SB strategy achieved a high, moderate, and low vaccination coverage in the three parishes, respectively. Notably children often brought dogs for vaccination indicating their relevant roles as reliable partners in dog vaccination campaigns. Sikana et al. established in their study that for the households which owned dogs, in 58% of them, it was the children responsible for taking dogs for vaccination (41). This resonates with experiences from the Namibia Rabies Elimination Program, where school-based education and active involvement of children were instrumental in increasing dog vaccination uptake and sustaining high coverage (42). The not constantly high performance was surprising, given that it was particularly rolled out in weekends when school children and their guardians were expected to be available to take dogs for vaccination. The low vaccination coverage among owned dogs in Kyatega Subcounty and in particular Kyatega parish can be explained by the dominant use of dogs for hunting activities, making them unavailable during vaccination exercise. For example, one evening while the team of researchers came from vaccination exercise, they met a dog owner with 17 dogs coming from hunting and all these dogs missed the vaccination.

The SP strategy performed poorly in this study. One reason can be the rather low number of vaccination points compared to the other strategies, leading to larger distances for dog owners to reach the sites. In the parishes Kisambya and Mpara Central, the model did not converge due to more dogs found vaccinated during the household survey in the involved villages, than had been recorded to be vaccinated during the vaccination campaign. This discrepancy points to the possibility of roaming dogs that crossover to different villages and homes. Another explanation may be a poor data quality both during the vaccination campaign and/or household survey. This strategy was chronologically the first one applied, so most prone for errors. Therefore, we could only estimate the vaccination coverage in one parish of the three, leaving the uncertainty about the performance and vaccination coverage reached for this strategy.

This study highlighted substantial differences in vaccination coverage among different parishes, ranging from 27.2 to 88.4% in owned dogs. Although the study aimed to explore the influence of the vaccination strategy on coverage reached, the within sub-county variation points out that other factors were influential. These include the differing sensitization and information campaigns, conduction of campaigns during weekdays versus weekends, or differing dog populations structures within people or dogs (for example some parishes with high proportion of refugees, varying density of dog owning households, varying dog:human ratios, different tribes, or presence of hunting dogs). Overall, the study could show that the three strategies that were so far not that often applied (SB, D-L, D-H) all performed well with vaccination coverages above 50% for all the parishes applied. It needs to be highlighted that the strategies selected for each sub-county was adapted to the respective setting, with the SB applied in a region with high density od schools, the D-L in the cattle corridor, and the D-H in a sub-county with active health centers. The study thus provided evidence for successful vaccination campaign when the performed strategies is adapted to the respective setting. Given the diversity of districts in terms of culture, dog ownership, and infrastructure, adapting strategies to local context for-example school based campaigns in areas with many schools or livestock campaigns in cattle corridors offers a practical pathway or national scale up.

An additional element that needs to be further investigated to inform future vaccination campaigns is the cost-effectiveness of the four campaign strategies. Public costs covered by governmental units on national ministry, district or sub-county level, need to be summed with private costs taken by dog owners, such as time and money spent to bring the dogs to the vaccination campaign to calculate the full cost of the campaign, and put it into relation with the number of dogs vaccinated (43, 44). Such an analysis was beyond the scope of this study. However it is planned to conduct a cost-effectiveness evaluation in combination with data from similar vaccination campaigns performed in another Ugandan district and publish them in a subsequent paper.

The findings of this study indicate that most dogs are owned, thus most dogs can be accessible once preliminary steps are carefully taken, such as sufficient mobilization, timely communication of the campaigns, and performance of free of charge services. Studies in Uganda and other African countries made similar observations, stating that most dogs in Africa are owned and accessible for vaccination, particularly when the vaccination is performed without costs for the dog owners (23, 34, 45–47). However, even amongst owned dogs, their accessibility by the owners, and the accessibility of dog owners themselves is not always given. During post vaccination evaluation, the vaccinators mentioned that in some parishes, like Kishagazi, dog owners were not accessible, despite the prior notices and mobilization for dog vaccination. This finding aligns with Kaneko et al., where low dog vaccination coverage was attributed to unavailability of dog owners at vaccination time (48). These cases highlight that for vaccination strategies focusing on owned dogs, information and motivation of owners is crucial for achieving a high vaccination coverage. The proportion of ownerless dogs estimated in this study range between 0.1–20.8%, with one outlier being Kijongobya parish with a proportion of ownerless dogs of 88.1%. The extremely large proportion of ownerless dogs is unclear and needs to be taken with caution. During interactions with stakeholders in this parish, they mentioned that there was a common practice by farmers who migrate to this area for agriculture. They often arrive with dogs to guard their crops after sowing. After harvesting, the dogs are left behind in the wilderness, while farmers return to their original locations, and become stray.

During this study, a substantially higher proportion of male dogs were vaccinated compared to females. This might be attributed on one hand to a higher population of male dogs kept by owners, driven by human preferences for specific tasks such as guarding and hunting that is perceived to be better performed by male dogs. Prior studies such as by Gibson et al. (49), Morters et al. (50), Davlin et al. (47), and Jibat et al. (23) have supported this observation, however only Jibat et al. (23) provided insights into reasons behind this occurrence, emphasizing the heightened demand for male dogs due to their aggressive nature and thus suitability for roles requiring security and hunting duties. On the other hand, this observation could have been because female dogs were less often presented at the vaccination points, either because they are not valued the same as males, or because of their reproductive cycles. During our vaccination campaigns, pregnant and lactating dogs were notably less presented for vaccination, a finding aligned with another study on barriers to attendance at static vaccination points in Malawi (51). Understanding these patterns is crucial for designing targeted and inclusive vaccination programs that address specific needs and barriers to improve overall vaccination coverage among different canine populations.

The study revealed that a significant majority of animals (up to 86.3%) were accompanied by adults when brought to vaccination sites, rather than children below 15 years of age. This is in contrast to other studies that reported a higher proportion of children bringing their dogs for vaccination (41). During consultations before the vaccination campaigns in Kyatega sub-county (SB strategy), stakeholders mentioned that their dogs are very aggressive and would pose a threat to children at the vaccination sites due to excitement from meeting other dogs. However, particularly for this sub-county that happened during weekends, the proportion of dogs brought by children was highest amongst all sub-counties. We thus more believe that the timing of the vaccination campaign during school days for the other three strategies contributed to less participation of children below 15 years.

Overall, this study comes with some limitations. First, the economic activities of Kyegegwa residents, particularly farmers leaving for gardens and crop fields with their dogs early and returning late, impacted the observations during transect walks and household surveys. Therefore, we likely observed fewer dogs than were actually there, which could affect our estimations. To overcome this limitation, transect walks were timed early in the morning and late in the afternoon, and household surveys were done around noon when farmers were anticipated to be back for lunch breaks in select villages. Secondly, the surveys (transect walks and household surveys) were conducted in villages with at least five dogs vaccinated during the vaccination campaign. This was done to reach a certain proportion of vaccinated dogs in the population to survey, to enable a suitable stability to the models. Although we believe that this approach mainly excluded villages with small dog populations, it might have led to an overestimation of the vaccination coverage in our study. Thirdly, roads in some of the areas got uncomfortable during the rainy season, therefore some of the study sites could not be accessed as had been planned. This was particularly obvious in Katamba parish. However, we did not observe a lower vaccination coverage in this parish compared to the other two parishes in the same sub-county, and thus we do not believe that this was a heavily impactful factor. Additionally, during estimation of dog population and vaccination coverage, we used priors from Chad, as we did not collect robust data from Kyegegwa to calculate them for our specific area- However, the priors were large enough so that it was still valid for our study area. Besides, the sensitivity analysis showed that the impact of the priors on the posterior estimates was negligible. Finally, we missed to collect gender-specific data on dog owners, depriving the study of potential insights to identify gender-specific compliance patterns for dog vaccination and to tailor approaches accordingly.

More broadly, the principle of integrating dog vaccination with existing community platforms is relevant across Africa, where resource constraints are common. Evidence from Chad, Tanzania and Malawi supports the potential transferability of such essential to establish their consistency, feasibility and cost-effectiveness for large application (52–54).

## Conclusion

The integrated D-H campaigns achieved the highest coverage among all strategies tested, demonstrating the strong potential of integrating animal and human health services to enhance community participation. Our study also showed the benefit of engaging the animal health officials as well as community representatives before conducting a vaccination campaign. This demonstrated the inherent complexity of implementing dog vaccination programs and the need for multifaced, context specific approaches. Future campaigns should be tailored to community needs, dog ownership patterns, and cultural values, while exploring integration with existing health services to maximize reach and impact. Further testing across diverse settings can help assess integrated dog vaccination strategies’ consistency and scalability, providing valuable insights for developing a One Health model to strengthen future rabies elimination efforts.

## Data Availability

The datasets presented in this study can be found in online repositories. The names of the repository/repositories and accession number(s) can be found in the article/Supplementary material.

## Footnotes

^1^ https://www.wvsapp.org/

^2^ https://www.mrc-bsu.cam.ac.uk/software/bugs-project

## References

[1] World Health Organization. Technical guidance for the prevention and management of rabies. Geneva: World Health Organization. (2023). Available online at: https://www.who.int/publications/i/item/who-wer9316.

[2] Hampson K Coudeville L Lembo T Sambo M Kieffer A Attlan M. Estimating the global burden of endemic canine rabies. PLoS Negl Trop Dis. (2015) 9:709. doi: 10.1371/journal.pntd.0003709, PMID: 25881058 PMC4400070

[3] World Health Organization. WHO Expert consultation on rabies. Geneva: World Health Organization (2018).

[4] Akusekera I Namayanja J Okello PE. *Dying rabid: adopting compulsory mass dog vaccination to reduce human deaths from dog rabies in Uganda: policy brief*. Uganda National Institute of Public Health, (2021).

[5] Masiira B Makumbi I Matovu JKB Ario AR Nabukenya I Kihembo C. Long term trends and spatial distribution of animal bite injuries and deaths due to human rabies infection in Uganda, 2001-2015. PLoS One. (2018) 13:e0198568. doi: 10.1371/journal.pone.0198568, PMID: 30130364 PMC6103508

[6] World Health Organization. *First Annual Progress Report United Against Rabies Collaboration*. Geneva: World Health Organization. (2021). Available online at: www.who.int.

[7] Duamor CT Hampson K Lankester F Lugelo A Mpolya E Kreppel K. Development, feasibility and potential effectiveness of community-based continuous mass dog vaccination delivery strategies: lessons for optimization and replication. PLoS Negl Trop Dis. (2022) 16:1–23. doi: 10.1371/journal.pntd.0010318 PMC9481168 36067231

[8] Changalucha J Steenson R Grieve E Cleaveland S Lembo T Lushasi K. The need to improve access to rabies post-exposure vaccines: lessons from Tanzania. Vaccine. (2019) 37:A45–53. doi: 10.1016/j.vaccine.2018.08.086, PMID: 30309746 PMC6863039

[9] Abubakar AT Al-Mustapha AI Oyewo M Ibrahim A Abdulrahim I Yakub JM. Prospects for dog rabies elimination in Nigeria by 2030. Zoonoses Public Health. (2024) 71:1–17. doi: 10.1111/zph.13084, PMID: 37933425

[10] Cleaveland S Thumbi SM Sambo M Lugelo A Lushasi K Hampson K. Proof of concept of mass dog vaccination for thecontrol and elimination of canine rabies. Rev Sci Tech. (2018) 37:559–68. doi: 10.20506/rst.37.2.2824, PMID: 30747125 PMC7612386

[11] Zinsstag J Lechenne M Laager M Mindekem R Naïssengar S Oussigéré A. Vaccination of dogs in an African city interrupts rabies transmission and reduces human exposure. Sci Transl Med. (2017) 9:6984. doi: 10.1126/scitranslmed.aaf6984, PMID: 29263230

[12] Kaare M Lembo T Hampson K Ernest E Estes A Mentzel C. Rabies control in rural Africa: evaluating strategies for effective domestic dog vaccination. Vaccine. (2009) 27:152–60. doi: 10.1016/j.vaccine.2008.09.054, PMID: 18848595 PMC3272409

[13] Sambo M Ferguson EA Abela-Ridder B Changalucha J Cleaveland S Lushasi K. Scaling-up the delivery of dog vaccination campaigns against rabies in Tanzania. PLoS Negl Trop Dis. (2022) 16:e0010124. doi: 10.1371/journal.pntd.0010124, PMID: 35143490 PMC8865671

[14] Léchenne M Oussiguere A Naissengar K Mindekem R Mosimann L Rives G. Operational performance and analysis of two rabies vaccination campaigns in N’Djamena, Chad. Vaccine. (2016) 34:571–7. doi: 10.1016/j.vaccine.2015.11.033, PMID: 26631415

[15] Fahrion AS Taylor LH Torres G Müller T Dürr S Knopf L. The road to dog rabies control and elimination-what keeps us from moving faster? Front Public Health. (2017) 5:103. doi: 10.3389/fpubh.2017.00103, PMID: 28555183 PMC5430047

[16] Mazeri S Burdon Bailey JL Mayer D Chikungwa P Chulu J Grossman PO. Using data-driven approaches to improve delivery of animal health care interventions for public health 118:e2003722118. doi: 10.1073/pnas.2003722118, PMID: 33468627 PMC7865124

[17] Evans MJ Burdon Bailey JL Lohr FE Opira W Migadde M Gibson AD. Implementation of high coverage mass rabies vaccination in rural Uganda using predominantly static point methodology. Vet J. (2019) 249:60–6. doi: 10.1016/j.tvjl.2019.04.013, PMID: 31239167

[18] Schelling E Bechir M Ahmed MA Wyss K Randolph TF Zinsstag J. Human and animal vaccination delivery to remote nomadic families, Chad. Emerg Infect Dis. 13:373–9. doi: 10.3201/eid1303.060391, PMID: 17552089 PMC2725911

[19] Lankester F Davis A Kinung’Hi S Yoder J Bunga C Alkara S. An integrated health delivery platform, targeting soil-transmitted helminths (STH) and canine mediated human rabies, results in cost savings and increased breadth of treatment for STH in remote communities in Tanzania. BMC Public Health. (2019) 19:1398. doi: 10.1186/s12889-019-7737-6, PMID: 31660915 PMC6819457

[20] World Organization for Anima Health. Trends in the use of veterinary vaccines: the case of rabies and PPR. Geneva: World Organization for Anima Health (2022).

[21] Wallace RM Undurraga EA Blanton JD Cleaton J Franka R. Elimination of dog-mediated human rabies deaths by 2030: needs assessment and alternatives for progress based on dog vaccination. Front Vet Sci. (2017) 4:9. doi: 10.3389/fvets.2017.00009, PMID: 28239608 PMC5300989

[22] Monje F Kadobera D Ndumu DB Bulage L Ario AR. Trends and spatial distribution of animal bites and vaccination status among victims and the animal population, Uganda: a veterinary surveillance system analysis, 2013–2017. PLoS Negl Trop Dis. (2021 Apr) 15:15(4). doi: 10.1371/journal.pntd.0007944, PMID: 33872314 PMC8084341

[23] Jibat T Hogeveen H Mourits MCM. Review on dog rabies vaccination coverage in Africa: a question of dog accessibility or cost recovery? PLoS Negl Trop Dis. (2015) 9:e0003447. doi: 10.1371/journal.pntd.0003447, PMID: 25646774 PMC4315526

[24] WOAH-Africa. *Uganda validates a national strategy on rabies elimination*. WOAH-Africa (2022).

[25] Republic of Uganda. *National Population and Housing Census 2024 Final Report*. Volume 1 (Main) Republic of Uganda. (2024).

[26] MoH. *Ministry of Health, Health Facility Master List*. (2018).

[27] World Health Organization. Bujubuli Health Centre Strives to offer the Best Health Services to Refugees in Kyaka II. Geneva: World Health Organization (2019).

[28] MOE&S. *Primary-Enlorment*. Report MOE&S (2021).

[29] MOE&S. *Government-secondary*. MOE&S. (2019).

[30] Kayali U Mindekem R Yémadji N Vounatsou P Kaninga Y Ndoutamia AG. Coverage of pilot parenteral vaccination campaign against canine rabies in N’djaména, Chad. Bull World Health Organ. (2003) 81:739–44. PMID: 14758434 PMC2572337

[31] R Core Team. R: A language and environment for statistical computing_. R foundation for statistical Computing. Vienna, Austria: R Core Team (2024).

[32] Durr S Mindekem R Kaninga Y Doumagoum Moto D Meltzer MI Vounatsou P. Effectiveness of dog rabies vaccination programmes: comparison of owner-charged and free vaccination campaigns. Epidemiol Infect. (2009) 137:1558–67. doi: 10.1017/S0950268809002386, PMID: 19327197

[33] Gelfand AE Smith AFM. Sampling-based approaches to calculating marginal densities. J Am Stat Assoc. (1990) 85:398. doi: 10.2307/2289776

[34] Mbaipago N Madjadinan A Amalaman DM Andrée Ndour P Zinsstag J Heitz-Tokpa K. General insights on obstacles to dog vaccination in Chad on community and institutional level. Front Vet Sci. (2022) 9:866755. doi: 10.3389/fvets.2022.866755, PMID: 36311655 PMC9597194

[35] Chevalier V Davun H Sorn S Ly P Pov V Ly S. Large scale dog population demography, dog management and bite risk factors analysis: a crucial step towards rabies control in Cambodia. PLoS One. (2021) 16:e0254192. doi: 10.1371/journal.pone.0254192, PMID: 34237103 PMC8266089

[36] Isiko J. Service-learning and community engagement yields benefits in zoonotic disease control: the case of rabies control in Mbuya II zone in Kampala, Uganda. Pan Afr Med J. (2017) 27:11. doi: 10.11604/pamj.supp.2017.27.4.12448

[37] Kessely H Revault D Zinsstag J Ouattara O Gbangou JB Wyss K. *September 2024 joint human and animal health campaigns in Chad*. (2024). Available online at: https://cabidigitallibrary.org.

[38] Belshaw Z Robinson NJ Dean RS Brennan ML. Motivators and barriers for dog and cat owners and veterinary surgeons in the United Kingdom to using preventative medicines. Prev Vet Med. (2018) 154:95–101. doi: 10.1016/j.prevetmed.2018.03.020 29685450

[39] Mulipukwa CP Mudenda B Mbewe AR. Insights and efforts to control rabies in Zambia: evaluation of determinants and barriers to dog vaccination in Nyimba district. PLoS Negl Trop Dis. (2017) 11:e0005946. doi: 10.1371/journal.pntd.0005946, PMID: 28991898 PMC5648261

[40] Hauskeller M. *Between the SpecieS living like a dog: can the life of non-human animals be meaningful?* Interne (2019). Available online at: http://digitalcommons.calpoly.edu/bts/.

[41] Sikana L Lembo T Hampson K Lushasi K Mtenga S Sambo M. Dog ownership practices and responsibilities for children’s health in terms of rabies control and prevention in rural communities in Tanzania. PLoS Negl Trop Dis. (2021) 15:e0009220. doi: 10.1371/journal.pntd.0009220, PMID: 33690720 PMC7946275

[42] Athingo R Tenzin T Shilongo A Hikufe E Shoombe KK Khaiseb S. Fighting dog-mediated rabies in Namibia—implementation of a rabies elimination program in the northern communal areas. Trop Med Infect Dis. (2020) 5:12. doi: 10.3390/tropicalmed5010012, PMID: 31963400 PMC7157552

[43] Mindekem R Lechenne MS Naissengar KS Oussiguéré A Kebkiba B Moto DD. Cost description and comparative cost efficiency of post-exposure prophylaxis and canine mass vaccination against rabies in N’djamena, Chad. Front Vet Sci. (2017) 4:38. doi: 10.3389/fvets.2017.00038, PMID: 28421186 PMC5376597

[44] Undurraga EA Millien MF Allel K Etheart MD Cleaton J Ross Y. Costs and effectiveness of alternative dog vaccination strategies to improve dog population coverage in rural and urban settings during a rabies outbreak. Vaccine. (2020) 38:6162–73. doi: 10.1016/j.vaccine.2020.06.006, PMID: 32616327

[45] Gsell AS Knobel DL Cleaveland S Kazwala RR Vounatsou P Zinsstag J. Domestic dog demographic structure and dynamics relevant to rabies control planning in urban areas in Africa: the case of Iringa, Tanzania (2012) 8:236. doi: 10.1186/1746-6148-8-236, PMID: 23217194 PMC3534358

[46] Wallace RML Mehal J Nakazawa Y Recuenco S Bakamutumaho B Osinubi M. The impact of poverty on dog ownership and access to canine rabies vaccination: results from a knowledge, attitudes and practices survey, Uganda 2013. Infect Dis Poverty. (2017) 6:97. doi: 10.1186/s40249-017-0306-2, PMID: 28569185 PMC5452361

[47] Davlin S Lapiz SM Miranda ME Murray K. Factors associated with dog rabies vaccination in Bhol, Philippines: results of a cross-sectional cluster survey conducted following the island-wide rabies elimination campaign. Zoonoses Public Health. (2013) 60:494–503. doi: 10.1111/zph.12026, PMID: 23280122 PMC3805987

[48] Kaneko C Omori R Sasaki M Kataoka-Nakamura C Simulundu E Muleya W. Domestic dog demographics and estimates of canine vaccination coverage in a rural area of Zambia for the elimination of rabies. PLoS Negl Trop Dis. (2021) 15:e0009222. doi: 10.1371/journal.pntd.0009222, PMID: 33909621 PMC8081203

[49] Gibson AD Handel IG Shervell K Roux T Mayer D Muyila S. The vaccination of 35,000 dogs in 20 working days using combined static point and door-to-door methods in Blantyre, Malawi. PLoS Negl Trop Dis. (2016) 10:e0004824. doi: 10.1371/journal.pntd.0004824, PMID: 27414810 PMC4945057

[50] Morters MK Mckinley TJ Restif O Conlan AJK Cleaveland S Hampson K. The demography of free-roaming dog populations and applications to disease and population control. J Appl Ecol. (2014) 51:1096–106. doi: 10.1111/1365-2664.12279, PMID: 25657481 PMC4285860

[51] Mazeri S Gibson AD Meunier N Bronsvoort BM d C Handel IG Mellanby RJ. Barriers of attendance to dog rabies static point vaccination clinics in Blantyre, Malawi. PLoS Negl Trop Dis. (2018) 12:e0006159. doi: 10.1371/journal.pntd.0006159, PMID: 29324737 PMC5783422

[52] Lechenne M Mindekem R Madjadinan S Oussiguéré A Moto DD Naissengar K. The importance of a participatory and integrated one health approach for rabies control: the case of N’Djaména, Chad. Trop Med Infect Dis. (2017) 2:43. doi: 10.3390/tropicalmed2030043, PMID: 30270900 PMC6082095

[53] Sánchez-Soriano C Gibson AD Gamble L Burdon Bailey JL Mayer D Lohr F. Implementation of a mass canine rabies vaccination campaign in both rural and urban regions in southern Malawi. PLoS Negl Trop Dis. (2020) 14:e0008004. doi: 10.1371/journal.pntd.0008004, PMID: 31971943 PMC6999910

[54] Duamor CT Hampson K Lankester F Lugelo A Changalucha J Lushasi KS. Integrating a community-based continuous mass dog vaccination delivery strategy into the veterinary system of Tanzania: a process evaluation using normalization process theory. One Health. (2023) 17:100575. doi: 10.1016/j.onehlt.2023.100575 37332884 PMC10272491

