# Supplementary material for: Comparing vaccination coverage and dog population demographics among four pilot dog rabies vaccination strategies in Uganda
Source: Front Vet Sci. 2025 Oct 13;12:1656563. doi: 10.3389/fvets.2025.1656563 (PMC12557789; doi:10.3389/fvets.2025.1656563)

Supplementary Material

Supplementary work for a study on Comparing vaccination coverage among four pilot dog rabies vaccination strategies in Uganda

*Supplementary Table S1: Number of dogs vaccinated per vaccination site in four sub-counties (Mpara, Kyatega, Ruyonza and Nkanja) and 12 parishes. Total number of dogs vaccinated = 850*

| *Parish* | ***Vaccination site*** | ***No. dogs vac/site*** | | ***Total no. dogs vac/parish*** |
| --- | --- | --- | --- | --- |
| Mpara Town Council: Static point (*SP), n= 152* | | | | |
| Bugido, 2 sites | Karokarungi | 41 | | 80 |
|  | Kyamutyetye | 39 | |  |
| Mpara central, 1 site | Mpara central | 28 | | 28 |
| Kisambya, 2 sites | Kyakikoyo and Kisambya | 44 | | 44 |
|  |  |  | |  |
| Kyatega sub-count**y***-School-based (SB), n= 292* | | | | |
| Katamba, 6 sites | Nyabitinda TC | 13 | | 53 |
|  | Kinyinya TC 0 | |  |  |
|  | Kyakikoba TC | 4 | |  |
|  | St. Claire P/S/Katamba TC | 22 | |  |
|  | Ibanda | 10 | |  |
|  | Kaziizi Rutoma | 4 | |  |
| Kyatega, 4 sites | Kyatega HQTrs | 41 | | 129 |
|  | Kicumu P/S | 32 | |  |
|  | Kisamula TC | 5 | |  |
|  | Kyangoma Church | 51 | |  |
| Nkomangani, 7 sites | Kabagara TC | 42 | | 110 |
|  | Kasenene P/S and Kasenene TC | 21 | |  |
|  | Kanyarukoma TC | 30 | |  |
|  | Nyarukoni | 3 | |  |
|  | Rurimengo | 7 | |  |
|  | Lukara TC | 7 | |  |
| Ruyonza sub-county: integrated dog with livestock vaccination strategy (D-L), *n=124* | | | | |
| Kishagazi, 2 sites | Kishagazi TC | 2 | | 20 |
|  | Mirembe | 18 | |  |
| Karwenyi, 2 sites | Karwenyi TC | 6 | | 26 |
|  | Katarubata | 20 | |  |
| Kijongobya, 4 sites | Kyakasamba, Kasikizi, Rwensasi | 70 | | 78 |
|  | Kijingobya TC | 8 | |  |
| Nkanja subcounty: integrated dog with human health services strategy (D-H), *n=282* | | | | |
| Kakoni, 3 sites | Kakoni B playground | 7 | | 38 |
|  | Kakoni C church | 12 | |  |
|  | Kakoni A church | 19 | |  |
| Bujubuli, 8 sites | Itambabiniga TC and Humura playground | 33 | | 214 |
|  | Bukere church | 0 | |  |
|  | Byabakoora TC | 61 | |  |
|  | Omururembo | 48 | |  |
|  | Ijugangoma | 49 | |  |
|  | Mukondo TC | 2 | |  |
|  | Sweswe TC | 21 | |  |
| Kyabirikuya, 2 sites | Kyayubu Catholic church | 30 | | 30 |
| Total |  | 850 | | 850 |

Supplementary Table S2: Prior distributions of the Bayesian model

| Parameter | Minimum value | Maximum value | Reference |
| --- | --- | --- | --- |
| Recapture probability | Uniform | Min = 0.056; max = 0.54 | Dürr et al, 2009 (zone 2) |
| Confinement parameter |  |  |  |
| Owned marked dogs | beta | α = 5.554; β= 13. 866  mean = 0.28, sd = 0.10 | Dürr et al, 2009 (zone 2) |
| Owned unmarked dogs | beta | α = 2.893; β= 11. 866  mean = 0.19, sd = 0.10 | Dürr et al, 2009 (zone 2) |

Supplementary Table S3: details of the findings from the household survey. The first column presents the number of dogs vaccinated during the campaigns in the villages where the household survey was conducted.

| **Parish** | **no. dogs vaccinated** | **no. households visited** | **no households with dogs** | **total. no of dogs in the households** | **no. roaming dogs** | **no. confined dogs in households** | **no. stray dogs reported by households** | **no. dogs seen on streets** | **no.dogs seen on compound** |
| --- | --- | --- | --- | --- | --- | --- | --- | --- | --- |
| Mpara Town Council: Static Point (SP) | | | | | | | | | |
| Mpara TC | 8 | 116 | 25 | 0 | 5 | 0 | 0 | 40 | 7 |
| Kisambya | 7 | 15 | 11 | 17 | 9 | 3 | 5 | 4 | 7 |
| Bugido | 39 | 29 | 25 | 43 | 23 | 0 | 10 | 6 | 21 |
| Kyatega: School-based (SB) | | | | | | | | | |
| Kyatega | 124 | 43 | 18 | 61 | 53 | 1 | 14 | 22 | 6 |
| Nkomangani | 95 | 35 | 4 | 8 | 13 | 1 | 6 | 17 | 10 |
| Katamba | 44 | 47 | 6 | 15 | 13 | 2 | 3 | 6 | 16 |
| Ruyonza: integrated dog with livestock vaccination strategies (D-L) | | | | | | | | | |
| Kisagazi | 18 | 40 | 5 | 7 | 7 | 0 | 5 | 8 | 8 |
| Karwenyi | 29 | 36 | 5 | 12 | 8 | 0 | 5 | 5 | 10 |
| Kijongobya | 42 | 83 | 19 | 35 | 25 | 0 | 14 | 14 | 30 |
| Nkanja: integrated dog vacciation with human health services (D-H) | | | | | | | | | |
| Bujubuli | 159 | 86 | 25 | 52 | 51 | 1 | 18 | 27 | 39 |
| Kakoni | 33 | 49 | 2 | 5 | 8 | 2 | 2 | 3 | 3 |
| Kyabirikuya | 28 | 29 | 8 | 19 | 19 | 0 | 1 | 3 | 22 |
| Total | **626** | **608** | **153** | **274** | **234** | **10** | **83** | **155** | **179** |

Supplementary Figure S1: Map showing selected sites from which we did transect walks for capture and recapture of dogs, as well as conducting household surveys. Right top corner is an extract Byabakoora transect


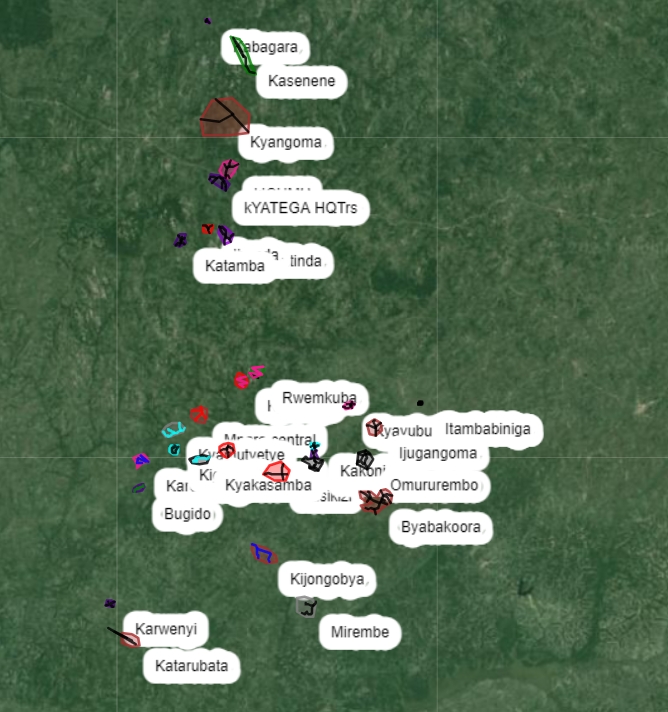


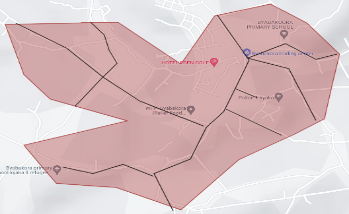


Supplementary Figure S1a: Map showing transect lines walked during the capture and recapture of dogs, as well as conducting household surveys.


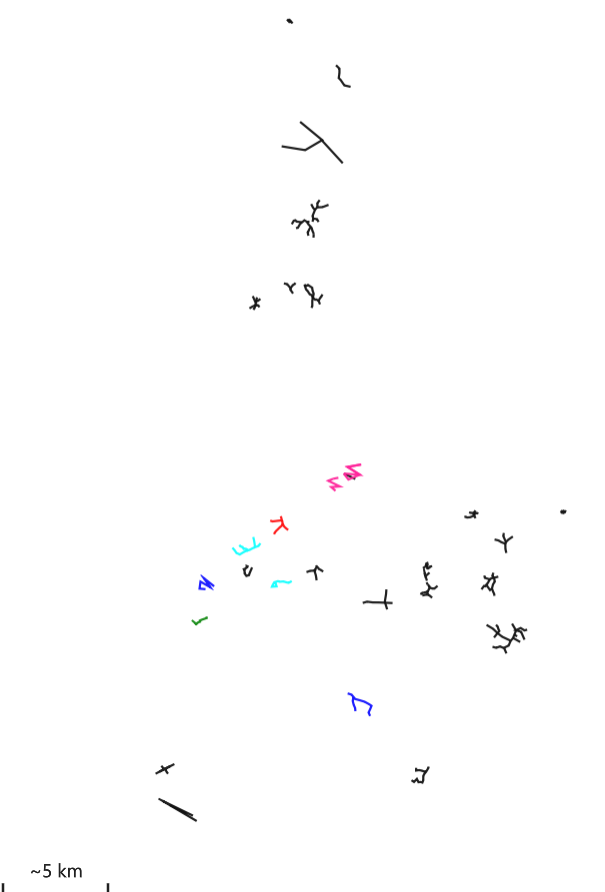


Supplementary Figure S1b: Map showing polygons for selected sites from which we did transect walks for capture and recapture of dogs, as well as conducting household surveys.


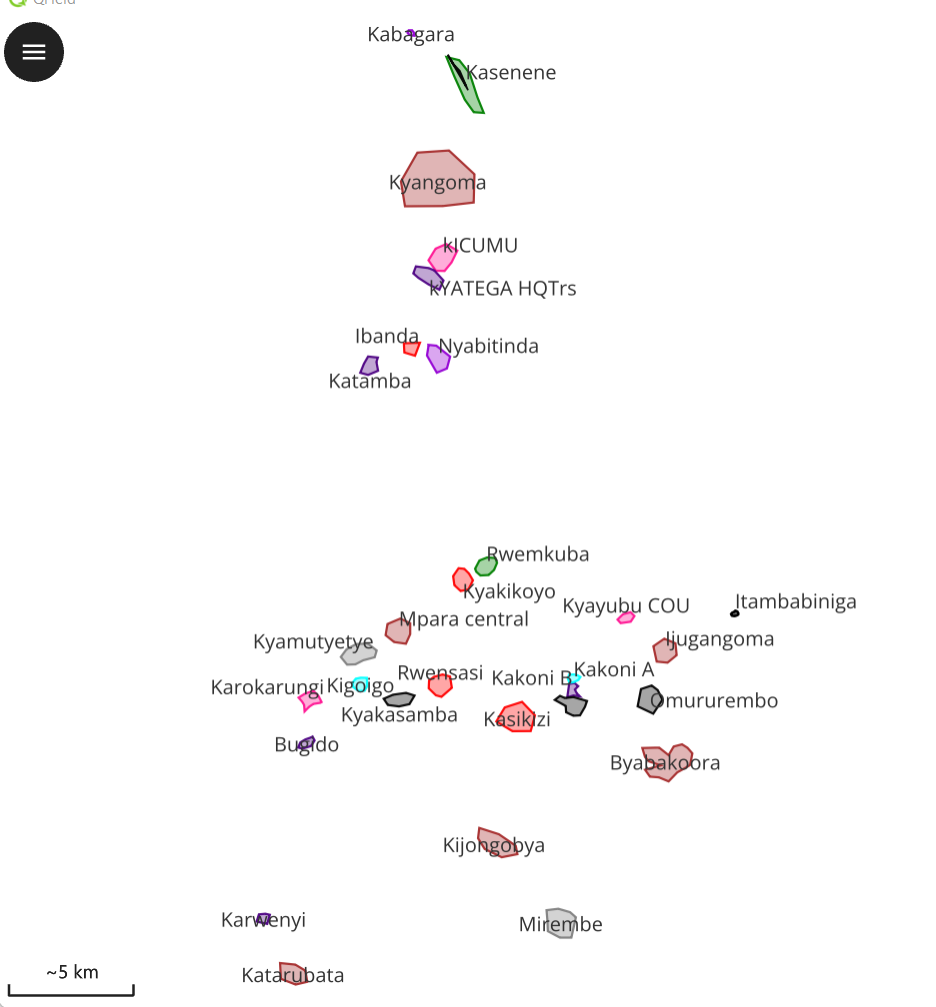


Supplementary Figure S2: A picture of mothers presenting their children at the human vaccination post during the integrated dog and human health services strategy in Kakoni parish, Nkanja subcountry, Uganda. Photo by:A research assistant Mr. Okello


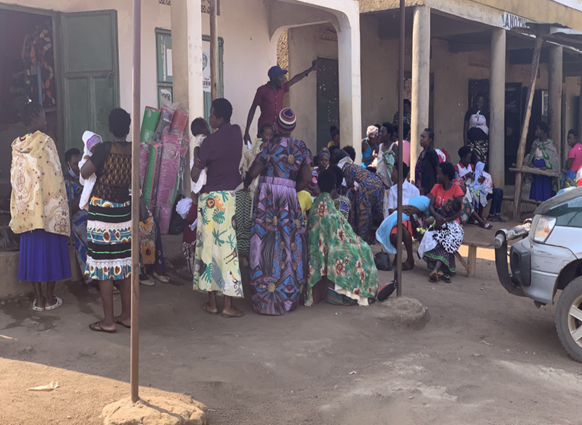


*Supplementary information 1*: Bayesian Model Code used for analysis

model {

for (i in 1:zone) {

for( t in 1 : T ) {

x1[i,t] ~ dbin(p[i,t],n1[i])

z[i,t] ~ dbin(p[i,t],n2[i])

x2[i,t] ~ dbin(p[i,t],n3[i])

p[i,t]~dunif(pmin[i],pmax[i])

}

n1[i]<-round((1-c1[i])*Mv[i])

n2[i]<-round((1-c2[i])*(M[i]-Mv[i])+aa[i]*M[i])

n3[i]<-round((1-c2[i])*(M[i]-Mv[i]))

logit(aa[i])<-a+rand[i]

c1[i]~dbeta(a1[i],b1[i])

c2[i]~dbeta(a2[i],b2[i])

cov_ow[i]<-Mv[i]/M[i]

cov_tot[i]<-Mv[i]/(M[i]+aa[i]*M[i])

M[i]~dnorm(Mmean[i],Mtau[i])

Mmean[i]<-Mv[i]*(peterson_n[i]+1)/(peterson_m[i]+1)

Mse[i]<-(pow(Mv[i],2)*(peterson_n[i]+1)*(peterson_n[i]-peterson_m[i]))/

(pow((peterson_m[i]+1),2)*(peterson_m[i]+2))

Mtau[i]<-1/sqrt(Mse[i])

a1[i]<-(m1[i]*m1[i]*(1-m1[i])/(s1[i]*s1[i]))-m1[i]

b1[i]<-a1[i]*(1-m1[i])/m1[i]

a2[i]<-(m2[i]*m2[i]*(1-m2[i])/(s2[i]*s2[i]))-m2[i]

b2[i]<-a2[i]*(1-m2[i])/m2[i]

rand[i]~dnorm(0,tau)

}

tau~dgamma(1,1)

sigma<-1/tau

a~dunif(-10,-2)

arat<-exp(a)/(1+exp(a))

}

From Transects

T: no of transects per zone

x1: no of captured marked owned (data collected in the transects)

z : no of captured unmarked (owned + ownereless) (data collected in the transects)

x2: part of z, number of owned unmarked dogs observed, latent factor (data cannot be observed)

pmin/pmax : prior parameters of a Uniform distribution for the recapture probabilities during transect

From vaccination campaign

Mv : total no of vaccined (marked + owned) dogs

From Household survey

peterson_n: total number of dogs counted during the household survey

peterson_m: number of marked dogs counted during the household survey

m1/s1: mean/sd of the prior distribution of the confinement probabililty (c1)

of marked (owned) dogs

m2/s2: mean/sd of the prior distribution of the confinement probabililty (c2)

of unmarked (owned) dogs

Latent factors that we want to estimate

M : total no of owned dogs (vaccinated/marked + unvaccinated)

aa: percentage for the calculation of ownerless dogs (= M*aa)

cov_ow: vaccination coverage within the owned dogs only

cov_tot: vaccination covergage within the total dog population (owned + unowned)

Data

Katamba

list(T=2,x1=structure(.Data=c(nb.marked morn,nb.marked eve.),.Dim=c(1,2)),z=structure(.Data=c(nb.unmarked mor,nb.unmarked eve),.Dim=c(1,2)),

zone=1,pmin=c(0.056),pmax=c(0.54),Mv=c(nb. vac.dogs),

m1=c(0.286),s1=c(0.1),m2=c(0.196),s2=c(0.1),

peterson_m=c(nb.marked HH),peterson_n=c(nb.total HH))

Bujubuli

list(T=2,x1=structure(.Data=c(14,17),.Dim=c(1,2)),z=structure(.Data=c(16,16),.Dim=c(1,2)),

zone=1,pmin=c(0.056),pmax=c(0.54),Mv=c(159),

m1=c(0.286),s1=c(0.1),m2=c(0.196),s2=c(0.1),

peterson_m=c(37),peterson_n=c(52))

Kakoni

list(T=2,x1=structure(.Data=c(1,0),.Dim=c(1,2)),z=structure(.Data=c(4,0),.Dim=c(1,2)),

zone=1,pmin=c(0.056),pmax=c(0.54),Mv=c(33),

m1=c(0.286),s1=c(0.1),m2=c(0.196),s2=c(0.1),

peterson_m=c(9),peterson_n=c(10))

Kyabirikuya

list(T=2,x1=structure(.Data=c(4,8),.Dim=c(1,2)),z=structure(.Data=c(6,6),.Dim=c(1,2)),

zone=1,pmin=c(0.056),pmax=c(0.54),Mv=c(28),

m1=c(0.286),s1=c(0.1),m2=c(0.196),s2=c(0.1),

peterson_m=c(12),peterson_n=c(19))

Kijongobya

list(T=1,x1=structure(.Data=c(9),.Dim=c(1,1)),z=structure(.Data=c(35),.Dim=c(1,1)),

zone=1,pmin=c(0.056),pmax=c(0.54),Mv=c(42),

m1=c(0.286),s1=c(0.1),m2=c(0.196),s2=c(0.1),

peterson_m=c(13),peterson_n=c(25))

Kishagazi

list(T=2,x1=structure(.Data=c(9,2),.Dim=c(1,2)),z=structure(.Data=c(7,2),.Dim=c(1,2)),

zone=1,pmin=c(0.056),pmax=c(0.54),Mv=c(18),

m1=c(0.286),s1=c(0.1),m2=c(0.196),s2=c(0.1),

peterson_m=c(4),peterson_n=c(7))

Karwenyi

list(T=2,x1=structure(.Data=c(0,9),.Dim=c(1,2)),z=structure(.Data=c(2,2),.Dim=c(1,2)),

zone=1,pmin=c(0.056),pmax=c(0.54),Mv=c(29),

m1=c(0.286),s1=c(0.1),m2=c(0.196),s2=c(0.1),

peterson_m=c(7),peterson_n=c(8))

Bugido

list(T=2,x1=structure(.Data=c(13,3),.Dim=c(1,2)),z=structure(.Data=c(7,9),.Dim=c(1,2)),

zone=1,pmin=c(0.056),pmax=c(0.54),Mv=c(39),

m1=c(0.286),s1=c(0.1),m2=c(0.196),s2=c(0.1),

peterson_m=c(3),peterson_n=c(13))

Mpara

list(T=2,x1=structure(.Data=c(0,3),.Dim=c(1,2)),z=structure(.Data=c(27,17),.Dim=c(1,2)),

zone=1,pmin=c(0.056),pmax=c(0.54),Mv=c(8),

m1=c(0.286),s1=c(0.1),m2=c(0.196),s2=c(0.1),

peterson_m=c(1),peterson_n=c(5))

Kisambya

list(T=2,x1=structure(.Data=c(6,0),.Dim=c(1,2)),z=structure(.Data=c(3,1),.Dim=c(1,2)),

zone=1,pmin=c(0.056),pmax=c(0.54),Mv=c(7),

m1=c(0.286),s1=c(0.1),m2=c(0.196),s2=c(0.1),

peterson_m=c(11),peterson_n=c(12))

Kyatega

list(T=2,x1=structure(.Data=c(3,2),.Dim=c(1,2)),z=structure(.Data=c(7,16),.Dim=c(1,2)),

zone=1,pmin=c(0.056),pmax=c(0.54),Mv=c(124),

m1=c(0.286),s1=c(0.1),m2=c(0.196),s2=c(0.1),

peterson_m=c(14),peterson_n=c(54))

Nkomangani

list(T=2,x1=structure(.Data=c(14,11),.Dim=c(1,2)),z=structure(.Data=c(2,0),.Dim=c(1,2)),

zone=1,pmin=c(0.056),pmax=c(0.54),Mv=c(95),

m1=c(0.286),s1=c(0.1),m2=c(0.196),s2=c(0.1),

peterson_m=c(7),peterson_n=c(14))

Katamba

list(T=2,x1=structure(.Data=c(6,4),.Dim=c(1,2)),z=structure(.Data=c(7,5),.Dim=c(1,2)),

zone=1,pmin=c(0.056),pmax=c(0.54),Mv=c(44),

m1=c(0.286),s1=c(0.1),m2=c(0.196),s2=c(0.1),

peterson_m=c(12),peterson_n=c(15))

Combined by strategy

School based

list(T=2,x1=structure(.Data=c(23,17),.Dim=c(1,2)),z=structure(.Data=c(16,21),.Dim=c(1,2)),

zone=1,pmin=c(0.056),pmax=c(0.54),Mv=c(263),

m1=c(0.286),s1=c(0.1),m2=c(0.196),s2=c(0.1),

peterson_m=c(33),peterson_n=c(83))

Integrated dog vs human health services

list(T=2,x1=structure(.Data=c(19,25),.Dim=c(1,2)),z=structure(.Data=c(26,22),.Dim=c(1,2)),

zone=1,pmin=c(0.056),pmax=c(0.54),Mv=c(220),

m1=c(0.286),s1=c(0.1),m2=c(0.196),s2=c(0.1),

peterson_m=c(58),peterson_n=c(81))

Integrated dog vaccination with live stock vaccination

list(T=2,x1=structure(.Data=c(18,11),.Dim=c(1,2)),z=structure(.Data=c(44,4),.Dim=c(1,2)),

zone=1,pmin=c(0.056),pmax=c(0.54),Mv=c(89),

m1=c(0.286),s1=c(0.1),m2=c(0.196),s2=c(0.1),

peterson_m=c(24),peterson_n=c(40))

Static strategy

list(T=2,x1=structure(.Data=c(19,6),.Dim=c(1,2)),z=structure(.Data=c(37,27),.Dim=c(1,2)),

zone=1,pmin=c(0.056),pmax=c(0.54),Mv=c(54),

m1=c(0.286),s1=c(0.1),m2=c(0.196),s2=c(0.1),

peterson_m=c(15),peterson_n=c(30))

Inits

list(

M = c(

330.3038445232613),

a = -9.356584917454322,

c1 = c(

0.6295521535442417),

c2 = c(

0.08356367020100683),

p = structure(.Data = c(

0.185840853427721,0.2465847272485976,0.2672889184446732,0.2603020433980903),

.Dim = c(1,4)),

rand = c(0.1312905972551651),

tau = 0.2606447148837419,

x2 = structure(.Data = c(39.0,57.0,65.0,59.0),

.Dim = c(1,4)))

list(

M = c(328.9904948690382),

a = -8.927177537206228, c1 = c(0.5564922341869767),

c2 = c(0.1142967926468905),

p = structure(.Data = c(0.141823363321519,0.2261289361018084,0.2270633341688325,0.2441727118316355),

.Dim = c(1,4)),

rand = c(0.8565254923147311),

tau = 1.491146880933921,

x2 = structure(.Data = c(34.0,56.0,35.0,58.0),

.Dim = c(1,4)))

**Supplementary Information 3: Ethical approval to conduct the study**


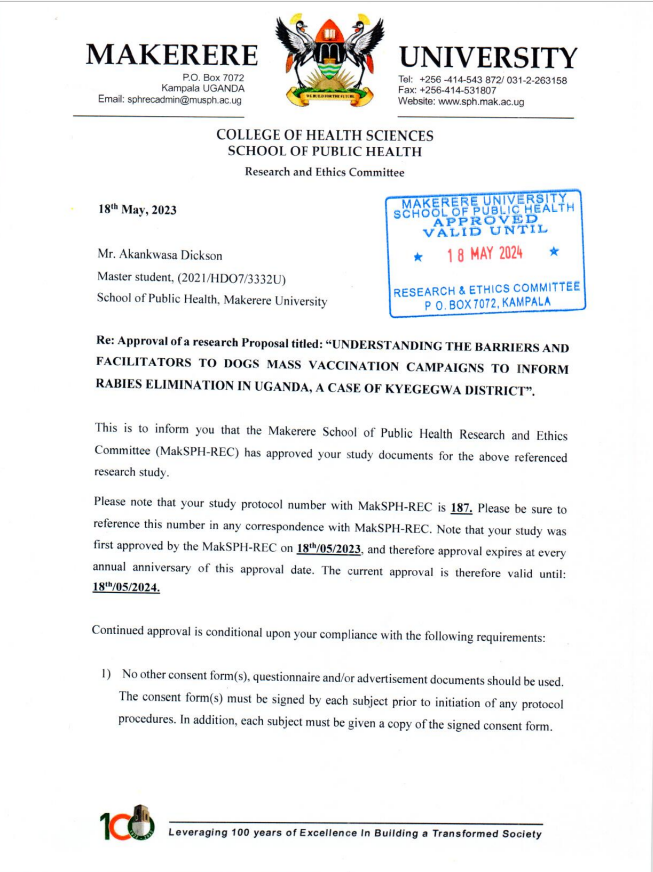


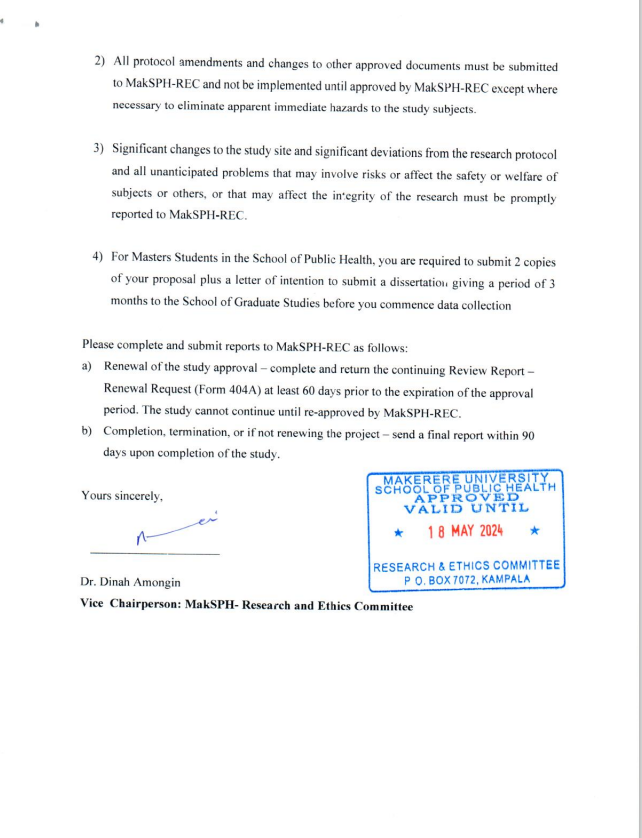


Supplementary Information 4: Permission and Kyegegwa district approval to conduct the study


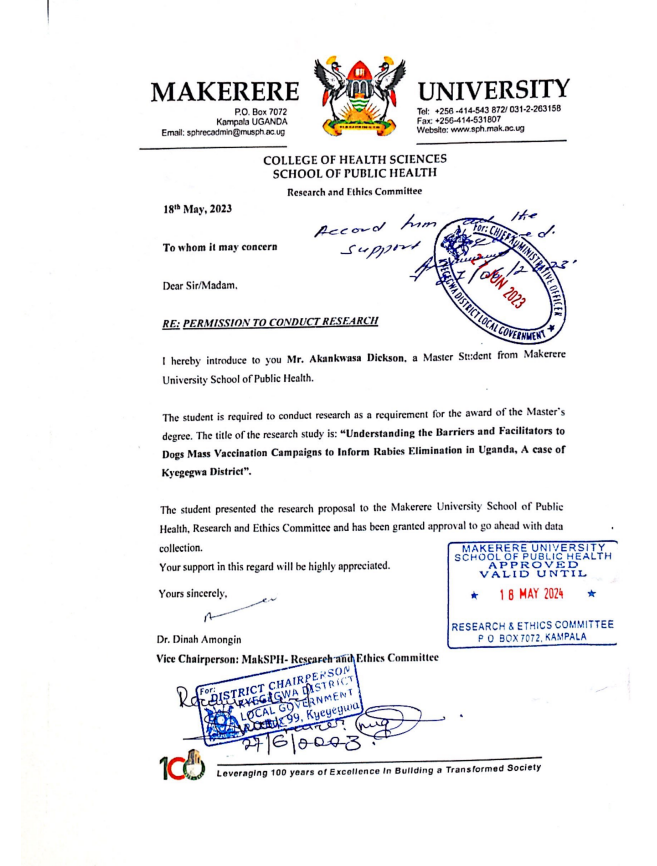

Supplement: Supplementary file 3 [file Data_Sheet_1.docx]
